# The role of AI in mitigating the impact of radiologist shortages: a systematised review

**DOI:** 10.1007/s12553-025-00970-y

**Published:** 2025-04-25

**Authors:** Nebil Achour, Tomas Zapata, Yousef Saleh, Barbara Pierscionek, Natasha Azzopardi-Muscat, David Novillo-Ortiz, Cathal Morgan, Mafaten Chaouali

**Affiliations:** 1 Faculty of Health, Medicine and Social Care, Anglia Ruskin University, Cambridge, UK; 2 World Health Organization Regional Office for Europe, Copenhagen, Denmark

**Keywords:** Artificial intelligence, Radiology workforce, Radiologist shortage, Legal and ethical considerations, Service resilience

## Abstract

**Purpose:**

This study aims to explore the application of Artificial intelligence (AI) systems in radiology departments and the role they play in the shortage of radiologists. It examines the ethical and legal considerations for uptake of AI both in relation to patient safety and for the profession of radiology.

**Methods:**

A systematised review was selected for this research study to collect maximum relevant evidence that provides a comprehensive overview of AI application in radiology specifically in terms of addressing radiologist shortages in hospitals. The search was complemented by grey literature to fill potential gaps.

**Results:**

Findings suggest that AI can read and interpret images more effectively and faster than radiologists and that it could be more widely used to reduce the impact of the global radiologist shortage, leading to better patient outcomes and safety. However, there are potential challenges predominantly ethical and legal. Concerns over complete radiologist replacement by AI do not currently seem likely, but rather the use of AI to complement radiologists in their work.

**Conclusions:**

AI cannot replace radiologists, instead radiology services will need the input of radiologists, AI systems and radiographers to provide a safe healthcare for all patients, therefore they are complementary. Radiologist jobs will most probably change to reduce repetitive tasks that can be conducted by AI. Radiologists and radiographers play a role in the provision of quality care in both normal day-to-day events and during times of disaster. Their role in diagnosing and prognosing diseases provides guidance during preparedness, response and recovery.

## Introduction

Technology’s prowess has been crystallised by global health emergencies, especially the COVID-19 pandemic. The healthcare landscape has been profoundly reshaped, bringing to light the pressing challenges such as critical shortages in specialised professionals [1], notably radiologists. This scarcity transcends merely numerical gaps [2]. It jeopardises patient safety and underpins clinical risk. Delays, misdiagnoses, and undesired patient outcomes have underscored an urgent call to innovation. Artificial Intelligence (AI) applications to radiology may appear to provide an innovative leap with the promise of strengthening health responses and patient care quality. However, contentious issues remain. These include how AI can effectively be used to address the radiologist shortage and concerns that AI might replace radiologists in hospitals as well as ethical and legal considerations including risk/benefit (non-maleficence) and autonomy of the patient and practitioner and accountability should AI make or deliver a misdiagnosis. There is little evidence to support a full understanding of these vital considerations. The aim of this study is to collect evidence to enable understanding of this problem in more detail and to suggest ways that support decision makers develop policies and strategies to protect radiology staff while applying AI technology and to set a research agenda to clarify unresolved issues. This study explores the technological aspects and delves deep into the ethical and legal terrains ensuring a holistic, patient-centric approach to AI adoption.

## Literature review

### Global radiologist shortage

Radiology has become a critical area of medicine, especially with the growing need to conduct an accurate diagnosis of a medical condition to facilitate treatment [3]. Radiology diagnostic tools are more common and popular for doctors than ever. The demand for Magnetic Resonance Imaging (MRI) and Computed Tomography (CT) scan services in England has been increasing by 10% each year since 2013 [4]. The United States of America (USA) had a consistent 30% growth in demand for radiologists since 2017 [5]. However, the number of radiologists has not increased adequately. The clinical radiology workforce in the United Kingdom (UK) grows by just 3% annually while demand for diagnostic activity is increasing by more than 5% [6]. Despite some signs of financial improvement due to high competition for staff [7], negative work-life balance, lack of continuous professional development and expected retirement of 20% of the current UK consultant radiologist workforce by 2028 all contribute to the shortfall of radiologists [8]. This shortage exposes patients to health deterioration; for example, 97% of UK cancer centre patients’ treatment was delayed due to staff shortages [9], and 48% of trusts and health boards have inadequate interventional radiologist (IR) services, meaning that only 34% of clinical directors felt they had enough IR to deliver safe and effective patient care [6]. An additional challenge in addressing the radiologist shortage is that it takes time for recent graduates to master reading diagnostic results. In the USA alone, there is expected to be a shortage of 122,000 radiologists by 2032. Additional reports have shown similar challenges in other countries, including in Poland, South Africa, Australia and Korea [10]. Only 2% of the UK radiology departments can meet imaging requirements in time [4]. This is even worse in low- and middle-income countries with limited staff and imaging resources [11].

### Vulnerability and need for innovation

**Fig. 1:**
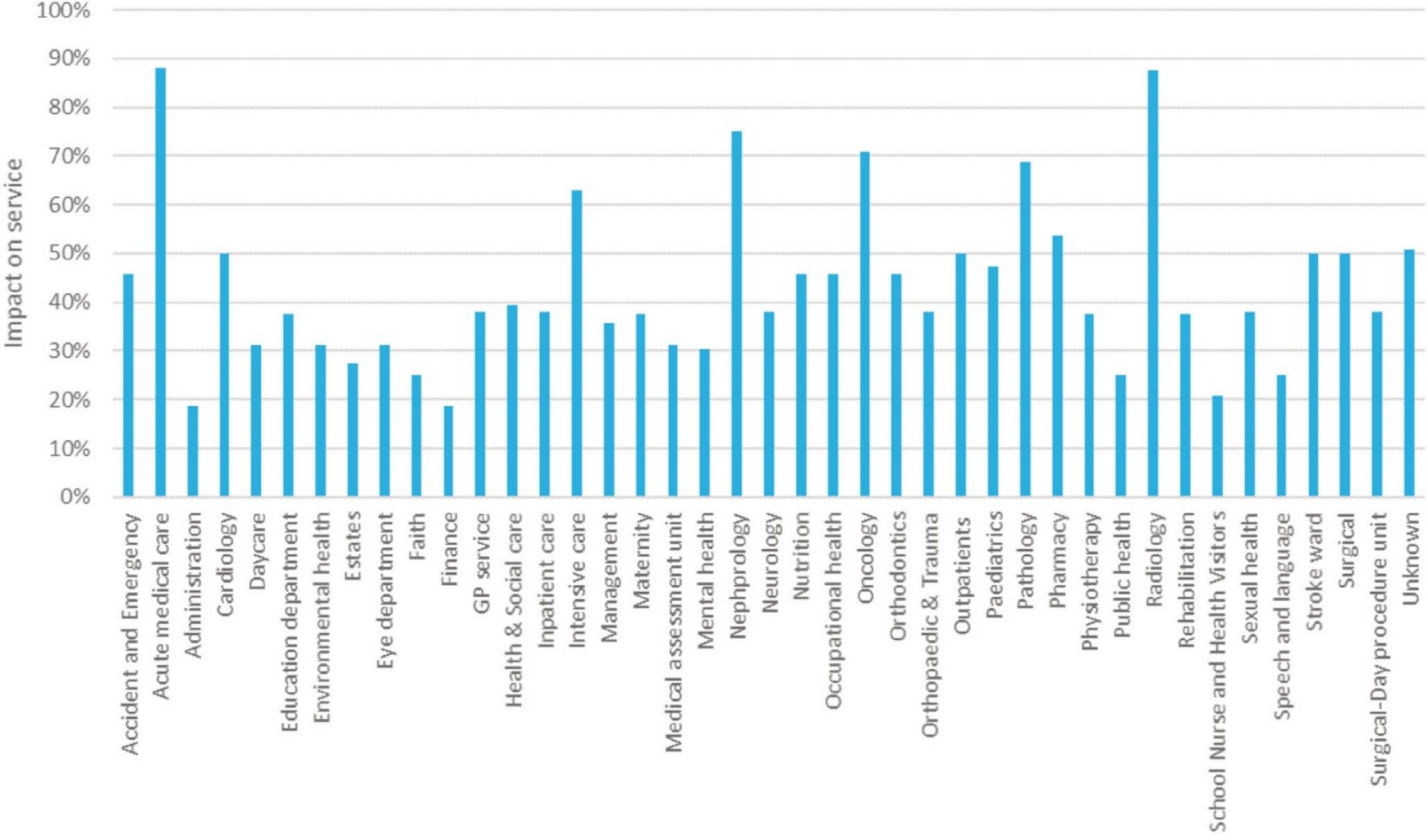
Radiology and acute medical care have the most significant impact on service (Achour et al., [13])

Indicators such as staff shortage and waiting lists, are a vulnerability of the health system and pose a risk to the continuity of healthcare service [12]. A health system is a series of systems that are highly interconnected and interdependent, the failure of any of these systems, or even one of their components will cause a domino-effect where other systems will fail to operate and cause a disruption within the health service. For example, many radiology departments operate with very limited staff members, the absence of one or two staff members during hazards and extreme weather events could lead to closure, see Fig. 1 [13]. The outbreak of the COVID-19 pandemic similarly put pressure on radiology departments globally denoting their vulnerability. Health systems were urged to adopt digital technology as part of their processes and diagnostic service such as measuring the maximum diameter of the aorta, and the volume of the heart with precision and efficiency.

AI brought a revolutionary innovation in diagnosis due to the generation of big data in the field of radiology and fuelled the argument that effectiveness of these machines is better than that of a human when it comes to doing repetitive tasks [14]. AI can interpret an MRI or CT-scan image better than a radiologist [15] which means that radiologists using AI systems can easily undertake and process a higher number of radiology tests [16] which reduces the pressure on radiologists and help cope better with the increasing demand. Several healthcare services in Europe and the USA were overstretched during the COVID-19 pandemic at a time of much greater demand. They rapidly implemented AI applications to X-ray and CT chest scans to reduce repetitive and time-consuming radiology tasks [16]. The impact of implementing AI varied in different countries, as some countries including the USA and Canada were already using this technology before the pandemic whereas others only introduced it during the pandemic. Countries with more limited resources are still without AI in their radiology departments [16]. This indicates that AI could help build greater resilience.

### The benefits of AI

AI has been introduced to radiology departments since the 1960s to diagnose [17]; however, computational limitations to process the poor quality of images limited their adoption in practice [18]. Modern AI is capable to read and interpret images efficiently and effectively and thus detect, predict outcomes such as pulmonary lesions and prostate cancer [19–22]. While radiologists have an average score of 66% accuracy in predicting brain tumour neuropathology, the score for AI is 85% [23]. Aortic analysis using AI can take two minutes, as opposed to 30 min using classical conventional methods, and can reduce the mean reporting time by 63% [24]. This technology therefore significantly reduces the time it takes to conduct a test, release a result, and interpret it in a way that is useful to physicians. In addition, using AI minimises human cognitive bias which can influence the effectiveness of radiologists [25]. These use of AI within radiology departments mean that the patient can be set upon the correct clinical pathway more quickly, ultimately resulting in improved patient safety and outcomes and reduced insurance costs [26]. The integration of AI and cloud systems has allowed radiologists to be available ‘anywhere, anytime’ which provides flexibility to radiologists and could be a reason to reduce emission associated with travel. A well-designed AI system can overcome the challenges associated with the use of IT multi-systems within a single department which radiologists are expected to manage. The types of radiology AI systems are expanding and healthcare providers are looking to get formal approvals from agencies such as the Food and Drug Administration (FDA) and Conformité Européenne (CE) [27].

## Methodology and protocol

### Data collection

A systematised review was selected for this research study to collect maximum relevant evidence that provides a comprehensive overview of AI application in radiology related to the shortage of radiologists. The search was complemented by grey literature to fill potential gaps. Data was collected from peer-reviewed journal articles. Databases including EMBASE, CINAHL Plus, MEDLINE, PubMed, and Cochrane Library were searched using keywords to identify relevant materials.

**Fig. 2:**
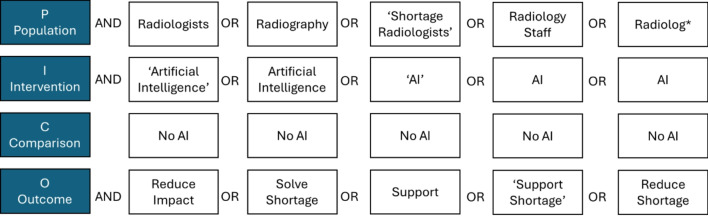
PICO process search selection 2017–2024

**Fig. 3:**
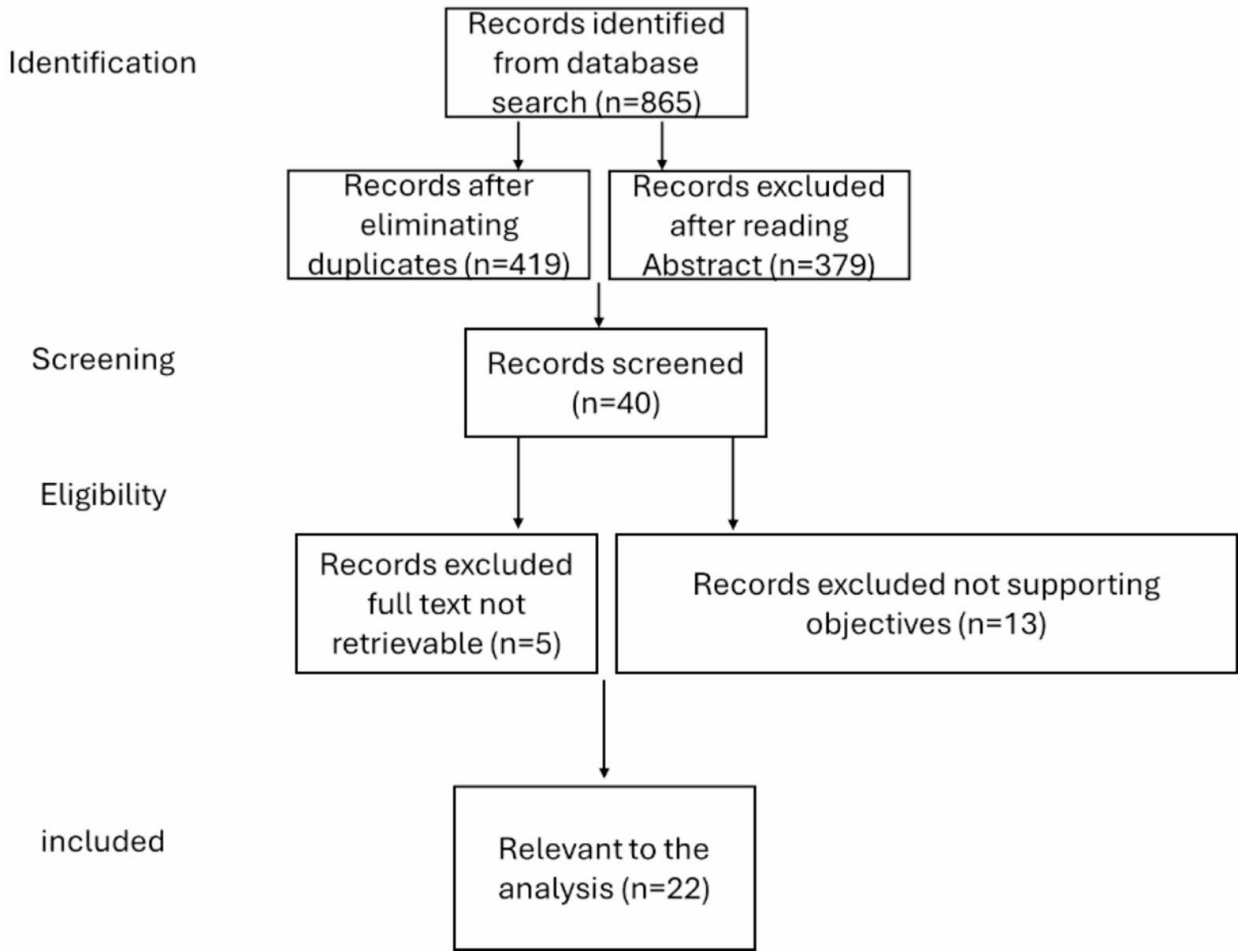
Prisma Flowchart

A Population, Intervention, Comparison and Outcome (PICO) process was applied to design the search keywords and identify precise search results (see Fig. 2). Inclusion and exclusion criteria were also applied to ensure that only data relevant to the goal of the study was obtained. Sources were also limited to publications both written in the English language and conducted between 2017 and (March) 2024. A Preferred Reporting Items for Systematic reviews and Meta-Analyses (PRISMA) flowchart (see Fig. 3) has been developed to illustrate the details of the search and selection of relevant data.

### Analysis

Narrative analysis was considered the right approach for this qualitative research. Out of the four narrative analyses, thematic analysis was the most appropriate to reach the objectives guided by the following framework:

- Explore the role of AI in mitigating radiologist shortages.
- Navigate the ethical and legal maze surrounding AI in radiology, highlighting concerns by different factors in the value chain.
- Provide recommendations to ensure AI’s application in radiology is ethically sound, legally compliant, and steadfastly patient-focused.

Different variables were considered when seeking data from identified sources. One variable was the characteristics of AI that make it possible to address the shortage of radiologists in hospitals. A second variable was the efficiency of the machines to perform the identified functions as effectively or better than a human would. A third variable was the quantity of work that AI can perform within a given period. Together these variables impact the speed at which AI can perform the tasks undertaken by radiologists, which could help to address the current problem of an overwhelming quantity of work for radiologists to perform.

## Findings

### Demographics

**Fig. 4:**
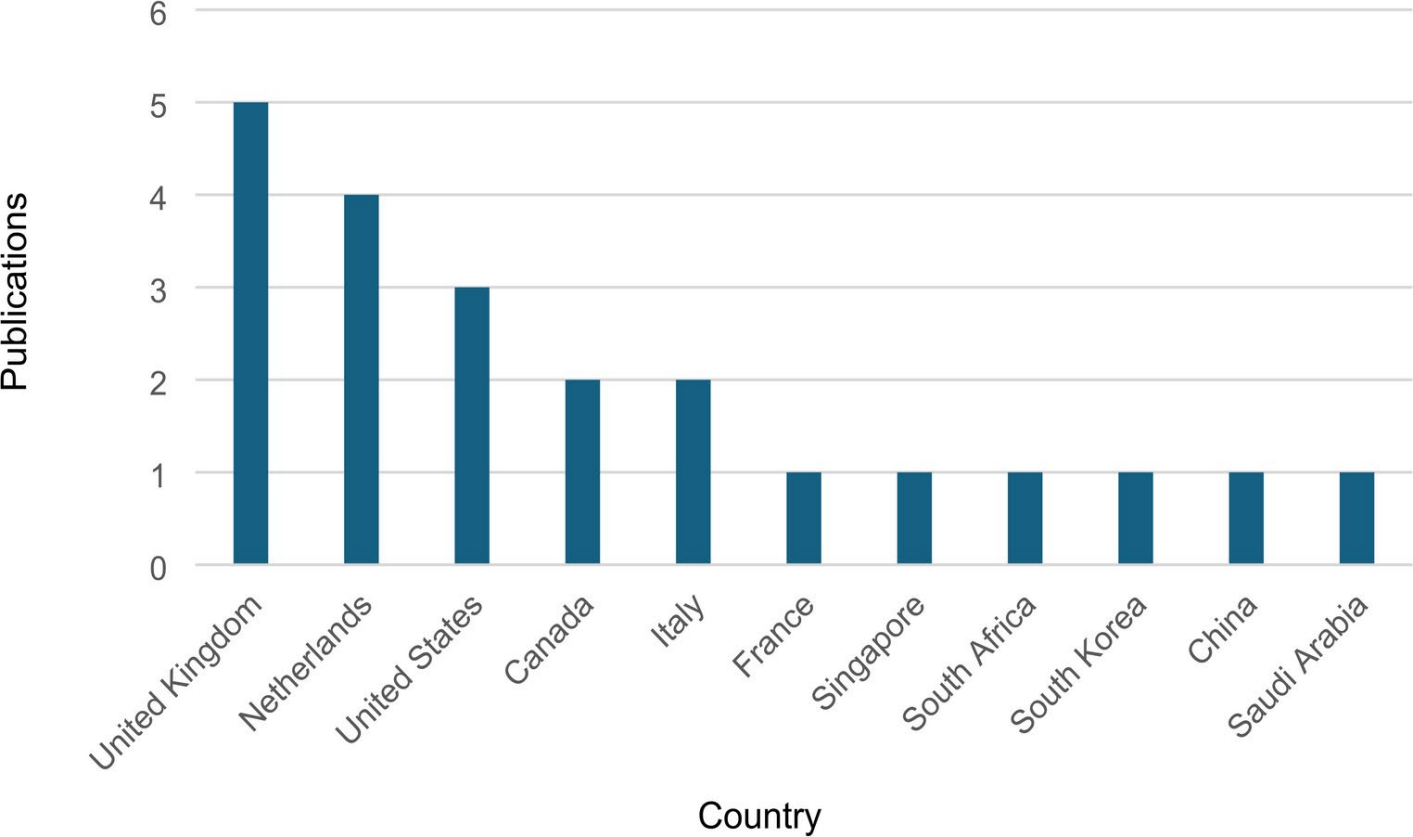
Publications per country

**Fig. 5:**
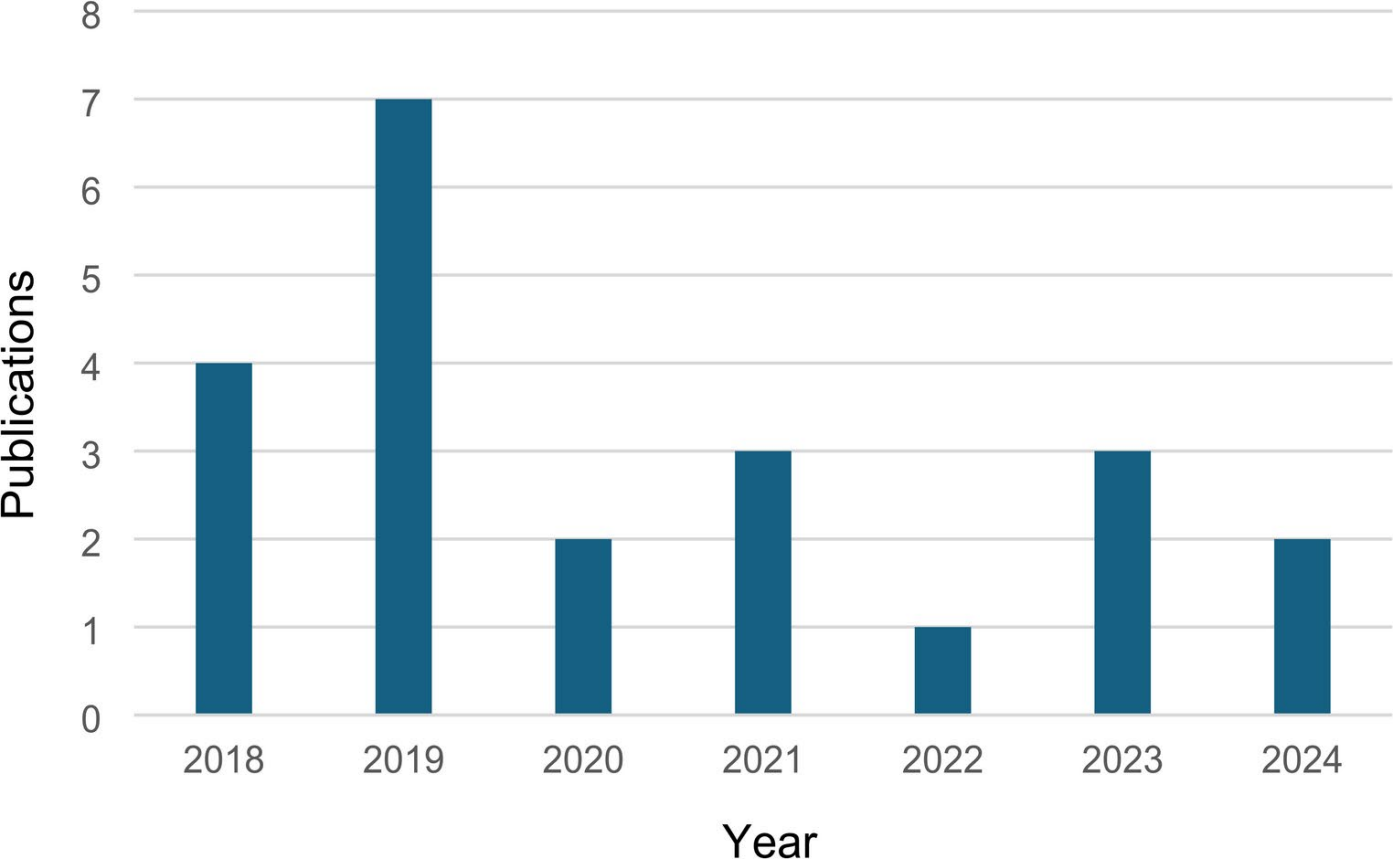
Publications per year

A total of 22 publications were found suitable for this study. These were predominantly led by researchers from the UK (*n* = 5), followed by The Netherland (*n* = 4) and the United States of America (USA, *n* = 3) see Fig. 4. Most of the sample were published in 2019 (see Fig. 5) and used secondary data (*n* = 12, 55%). Primary data (*n* = 9, 41%) and opinions (*n* = 1, 4%) were also identified. Approximately 55% of the studies are qualitative, followed by quantitative (*n* = 9, 41%) and mixed method (*n* = 1, 4%) denoting that the topic is still in its infancy and thus expected to grow in the future (see Table 1).

**Table 1:** Data extraction

Authors	Year	Country	Data	Study Design
Kalidindi, and Gandhi [8]	2023	United Kingdom	Secondary	Mixed
Ahuja [28]	2019	United States	Secondary	Qualitative
Hardy, and Harvey [29]	2020	United Kingdom	Secondary	Qualitative
He, et al. [30]	2019	China	Secondary	Qualitative
Hosny, et al. [31]	2018	United States	Opinion	Qualitative
Shaheen [32]	2021	Saudi Arabia	Secondary	Qualitative
Kwee and Kwee [33]	2021	The Netherlands	Secondary	Quantitative
Rodriguez-Ruiz, et al. [34]	2019a	The Netherlands	Primary	Quantitative
Syer, et al. [35]	2021	United Kingdom	Secondary	Quantitative
Rodriguez-Ruiz et al. [36]	2019b	The Netherlands	Primary	Quantitative
Waymel, et al. [37]	2019	France	Primary	Quantitative
Pesapane, et al. [38]	2018	Italy	Secondary	Qualitative
Neri, et al. [39]	2020	Italy	Secondary	Qualitative
Liew [40]	2018	Singapore	Secondary	Qualitative
Rodríguez-Ruiz, et al. [41]	2018	The Netherlands	Primary	Quantitative
Geis, et al. [42]	2019	United States	Primary	Qualitative
Gong, et al. [43]	2019	Canada	Primary	Quantitative
Sihlahla, et al. [44]	2023	South Africa	Secondary	Qualitative
Doria [45]	2023	Canada	Primary	Qualitative
Rainey, et al. [46]	2022	United Kingdom	Primary	Quantitative
Park, et al. [47]	2024	South Korea	Primary	Quantitative
Sangvik Grandal, et al. [48]	2024	United Kingdom	Secondary	Qualitative

### Data synthesis

Researchers have dedicated a substantial effort developing knowledge around radiology staff shortage and ethical and legal considerations. These have been summarised into key statements and included in Table 2. A major proportion of the research work has been dedicated to identifying how AI supports staff with a lower dedication to how AI systems can play a role in augmenting or reducing the shortage of radiologists, see Tables 2 and 3. Ethical and legal considerations were considered mostly on data and information management (11/22 studies) with little attention given to staff protection (3/22 studies), risk/benefit analysis for patients or arguments on whether AI presents an opportunity or threat to radiologists and radiographers. Findings also established that staff shortage was investigated from two perspectives, AI support (20/22 studies) and the role of AI in the shortage (8/22 studies), see Table 3. Considering that most of these studies are qualitative and based on secondary data, it might be prudent to say that the body of knowledge has not matured yet and that there is need for more work to be done to understand the implications of AI on the radiologist shortage.

**Table 2:** Extracted study key findings

Authors	Key findings
Kalidindi, and Gandhi [8]	• AI can be used as one way to address the radiologist shortage in UK and should be rapidly adopted. • AI benefits include improving efficiency and patient safety. • AI can be used across patient journey: radiological investigation request, safe image acquisition, alerting staff about critical and life-threatening situations, cancer screening follow up, generating radiology reports efficiently.
Ahuja [28]	• AI supports the work of radiologists by reducing the amount of work human experts are expected to undertake. • AI identifies the potential for unethical usage of personal health data in AI development.
Hardy and Harvey [29]	• The use of AI creates autonomy in running the machines, making it possible to conduct more tasks at a reduced cost and with minimal human expertise.
He, et al. [30]	• Although AI has numerous benefits, including the reduced number of radiologists needed at a given health centre, authorities must regulate its application to reduce risks.
Hosny, et al. [31]	• AI enhances image-based tasks in hospitals when handling cancer patients. • It is necessary to address challenges faced in the clinical implementation of AI in such settings. • AI “is unlike human intelligence in many ways; excelling in one task does not necessarily imply excellence in others”.
Shaheen [32]	• AI can re-balance workload to allow more interaction with patients. • AI can also help in reducing the cost of operation in the long term because of the speed and the reduced need for human experts.
Kwee and Kwee [33]	• AI is regarded as able to reduce the workload of radiologists, but it is likely to increase causes fragmentation in the workflow which will cause higher workload.
Rodriguez-Ruiz, et al. [34]	• AI has the capability to significantly reduce breast cancer screening reading workload by excluding exams of low cancer risk.
Syer, et al. [35]	• There is insufficient evidence to recommend clinical deployment of AI-based computer-aided diagnosis (CAD) systems for initial prostate cancer diagnosis.
Rodriguez-Ruiz et al. [36]	• AI enhances cancer detection accuracy because of its capacity to read and interpret numerous variables within a relatively short period. • AI technology is still new and needs further investigation before it can be adopted in radiology.
Waymel, et al. [37]	• Radiologists are optimistic that AI is going to bring positive changes in their field of study. • A significant number of those interviewed had limited knowledge about its application in this field, however they are willing and determined to learn more about it.
Pesapane, et al. [38]	• The AI legal framework is unclear, especially with the growing concerns about data privacy policies both in the United States and Europe. When these concerns are effectively addressed, AI will become an integral part of radiology.
Neri, et al. [39]	• The overall responsibility associated with AI application in diagnosis, and accuracy of its results, are yet to be legally determined. Although this technology has obvious benefits, it is still unclear how some of its shortcomings should be addressed and who should be held accountable if it makes major mistakes.
Liew [40]	• Clinical radiology is increasingly getting augmented with AI, which reduces the demand for radiologists in hospitals. • There are legal and ethical issues that still need to be addressed to effectively integrate AI in practice. • AI has the potential for job displacement (not necessarily replacement) within radiology.
Rodríguez-Ruiz, et al. [41]	• The sensitivity and accuracy of digital mammographic examinations increase significantly when AI is used. There is a clear and direct relationship between accuracy in mammographic examinations and the use of AI. • The technology should be embraced to help in the screening of women for breast cancer.
Geis, et al. [42]	• AI has become an integral part of radiology and many other aspects of the medical system. However, ethics in AI remains a major concern. Defining who is credited with the successes of AI and those who should be blamed for its mistakes remains a divisive issue.
Gong, et al. [43]	• Medical students shared their concern with the application of AI in radiologists. AI has the potential to mitigate the shortage of radiologists, but the concern is that the development of the technology might lead to reduced demand for radiologists. • Medical students are anxious about their replacement as AI accuracy increases, the need for radiologists will decrease.
Sihlahla, et al. [44]	• AI-enhanced health technologies within radiology will increase workflow efficiency, improve diagnostic accuracy, reduce healthcare-related costs, alleviate medical staff shortages. • Identifies four legal and ethical issues: AI transparency and informed consent, data protection, non-maleficence: liability, accountability, justice and fairness. • Recommendations for radiology practitioners using AI in South Africa are vigilant monitoring of AI use in practice, reforms to the liability framework, appropriate ethical guidance from local regulators and the Health Professions Council of South Africa.
Doria [45]	• Challenges to utilising AI are patient safety, data sharing and privacy regulations, workforce education and future jobs shortage, different perspectives on usage, healthcare inequity. • There is a need for big data, data sharing agreements, data standardisation, data integration into workflows
Rainey, et al. [46]	• Radiographers feel less confident in explaining how AI makes decisions to colleagues, patients, and carers, and believe that clinicians (radiologists) are in better position to do so.
Park, et al. [47]	• AI was effective in quickly and accurately diagnosing and classifying staging of liver fibrosis supporting radiologists. • AI can be adopted in radiology departments, further improvement and validation are however still required. • AI could help alleviate the radiologist shortage especially in low to middle income countries.
• Sangvik Grandal, et al. [48]	• AI is promising, but barriers to AI implementation are current healthcare infrastructure, ethical, legal and regulatory concerns, quality of AI algorithms, cost of implementation. • AI currently lacks clinical knowledge of radiologists and sometimes limited by practical barriers e.g. patient positioning. • Attitudes of radiologists and patients can be distrustful of decisions made by AI in part due to negative media portrayal.

**Table 3:** Data evaluation

Authors	Year	Staff shortage		Ethical and legal considerations	
		AI role in the shortage	AI support	Data and information management	Staff protection
Kalidindi, and Gandhi [8]	2023	x	x	-	-
Ahuja [28]	2019	x	x	x	-
Hardy, and Harvey [29]	2020	-	x	-	-
He, et al. [30]	2019	-	x	-	-
Hosny, et al. [31]	2018	-	x	x	-
Shaheen [32]	2021	-	x	-	-
Kwee and Kwee [33]	2021	x	x	-	-
Rodriguez-Ruiz, et al. [34]	2019a	-	x	-	-
Syer, et al. [35]	2021	-	x	-	-
Rodriguez-Ruiz et al. [36]	2019b	-	x	x	-
Waymel, et al. [37]	2019	-	x	-	x
Pesapane, et al. [38]	2018	-	-	x	x
Neri, et al. [39]	2020	x	x	x	x
Liew [40]	2018	-	x	x	-
Rodríguez-Ruiz, et al. [41]	2018	-	x	-	-
Geis, et al. [42]	2019	-	-	x	-
Gong, et al. [43]	2019	x	x	-	-
Sihlahla, et al. [44]	2023	-	x	x	-
Doria [45]	2023	x	x	x	-
Rainey, et al. [46]	2022	x	x	x	-
Park, et al. [47]	2024	x	x	-	-
Sangvik Grandal, et al. [48]	2024	-	x	x	-

### Role of AI in mitigating the radiologist shortages

The shortage of workforce in radiology has been noticed for many years. It became more apparent after the Covid-19 pandemic. Countries such as the UK had a shortfall of 33% in the radiology workforce in 2023 and that was set to reach 44% by 2024 and is expected to adversely affect the effectiveness of healthcare service provision and patient safety [9]. There is a common belief amongst researchers that AI supports staff in various ways, specifically in repetitive tasks, which tend to cause pressure and consume significant amounts of time [28, 29]. Hosny et al. [30] reported that an average radiologist is expected to interpret an image every 3 to 4 seconds which when put in context of a typical 8-hour working day will inevitably lead to errors, poor decisions and burnout. Setting AI to recognise and eliminate the high and low likelihood threshold will reduce the number of exams to be conducted by the radiologists which can reduce workload to approximately 53% [31–33] but can increase fragmentation in the workflow which might increase workload [32]. Ahuja [28] supported this opinion indicating that AI systems perform automatic segmentation that enables them to isolate and identify pathologic lesions that will reduce the examination time of the radiologists and thus reduce their workload and risk of error. Rodriguez-Ruiz et al. [34] and Syer et al. [35] went further by indicating that radiologists might not be needed in examinations that involve low and high probability of diagnosing an illness due to the high sensitivity of AI. AI systems sensitivity is comparable to experienced radiologists and can provide a good opportunity to reduce errors due to fatigue and distraction as well as boosting the sensitivity of less experienced radiologists [35]. A survey of 270 radiologists showed that only 52% believe that this freed time will be dedicated to spending it with patients which might explain the current level of overwork staff experience; although 81% (*n* = 219) expect AI to lower imaging-related errors; 73% (*n* = 214) believe that AI will have a positive impact on their jobs, 74% (*n* = 201) anticipate lower interpretation time and thus workload [36]. This support however is not convincing to students of radiology due to the unknown impact of AI on their career; approximately one in six (17%) interest medical students are discouraged from radiology solely [37]. Distrust in AI is likely to continue for some time. He et al. [38] and Rodriguez-Ruiz et al. [33, 39] confirmed the high sensitivity of AI and its ability to increase efficiency [40] but discouraged too much trust in this high sensitivity of AI solely until regulations have been sufficiently updated to effectively deal with the consequences should these systems fail, and that monitoring and vigilance need to fully be applied.

Non-academic discussions have been fuelling the possibility that AI will replace radiologists which might have caused lack of motivation of young doctors to follow careers in radiology [37, 41]. Park et al. [42] supports this opinion by stating that AI could alleviate radiologist shortages specifically in low- and middle-income countries. However, researchers such as Neri et al. [41] argued that AI cannot replace radiologists and that AI tasks should be implemented in radiology as an approach “to compensate for the progressive lack of radiologists” confirming that the use of AI reduces the number of repetitive image examinations and readings. Accountability, human oversight and understanding of how decisions are made, and privacy and data protection are some of the requirements that AI needs to meet before it becomes trustworthy and able to ‘replace’ humans in radiology departments [41]. Researchers, however, suggest that radiologists who resist learning and operating AI might be replaced with radiologists who do [43]. This indicates a potential for change in the job description rather than replacement [43–45]. This change is what has been referred to as job ‘displacement’ where roles change to combine both AI and radiology jobs e.g. ‘radiomics’ and ‘radiogenomics’ [45] and might escalate to create new areas within the professional organisations such as Data Science Institute by the American College of Radiology [43]. Such change cannot take place until radiologists become more involved in the development of the AI systems to understand and influence how decisions are made and are able to effectively evaluate AI tools and models prior to implementation as well as understand shortfalls and limitations of their outputs [37, 43]. This will also entail the willingness of health services, such as the UK National Health Service (NHS), to integrate these systems in assisting with diagnosis to reduce pressure on the radiology workforce [46].

The future operation of radiology services will depend on three key components: radiologists who as medical professionals are responsible for clinical decisions and for explaining findings, radiographers who provide technical support services and may well utilise AI and IT systems that can provide evidence for informed decision making. These components need to be reviewed and carefully integrated to ensure where accountability lies if AI is to have the potential to ease the radiology workforce crisis without threatening their roles and existence [8].

### Accountability for decisions

Researchers such as Pesapane et al. [44] suggest that radiologists have been pioneers in the application of digital medicine for a very long time and that the challenge in implementing AI does not come from them but from the regulatory institutions and authorities who question accountability for errors. Humans only consider the most obvious when making decisions, whilst AI systems conduct complex calculations that consider various eventualities which may render their decisions unpredictable [30, 44]. This raises the concern of who is responsible when these decisions are wrong, the developer, the radiologist or the machine. Young medical students believe that the responsibility should be with radiologists (60%) or shared between the radiologists and the developing company (35%) [36]. The identification of this responsibility will help in correcting the consequence of the mistake and the potential harm. Minimising harm or non-maleficence is one of the key ethical principles in medicine and biomedicine [47] in addition to respect for autonomy [29], beneficence and confidentiality [47]. Geis et al. [48] suggested that the radiology community should develop AI codes to promote legal use of data that befits patients and the common good and to prevent its use for financial interest.

Key legal concerns are associated with the application of AI in radiology departments, namely safety, failure judicial transparency and privacy [45]. Data protection regulations require robust and secure infrastructure, processing and storage [49].

## Discussion

The shortage of radiologists is a global concern that can have significant impact on the provision of health service and the ability to threaten the lives of many people specifically with increasingly complex and serious illnesses such as cancer. Problems such as workload and burnout are associated with errors and misdiagnoses which reduce the quality of care, increase unnecessary work or overlook the severity of serious health conditions for some patients. This has been raised as a concern and vulnerability of health systems and required urgent interventions to mitigate risks by reducing radiologist ramifications. AI has been present for many decades; however, in recent years, with the significant development of computing ability, AI has become one of the key innovations within radiology with the potential to improve health responses and patient care quality and resilience due to its ability to read and interpret images, videos and audios to generate evidence that can enable quicker and potentially more accurate decisions. However, there are many obstacles associated with the implementation of AI in radiology departments specifically those related to its ability to reduce the shortage of radiologists, and the ethical and legal consequences when errors and mistakes are identified.

### Ability of AI to reduce the shortage of radiologists

The literature revealed that there is consistency between researchers that AI can assist in reducing the workload of radiologists. The contrast however is between how researchers view this assistance. AI systems have developed very high sensitivity, to the extent that they can replace radiologists in specific decisions where the probability of being ill is very little or when it is almost certain that the patient is diagnosed with a particular illness such as cancer. Despite the high sensitivity, there is a risk that this diagnostic is not accurate and thus someone needs to take the responsibility when there is an error. This is one of the debates of this opinion. The other opinion is about trusting the AI to generate evidence and judgement under the full supervision of radiologists which will leave the decision and responsibility with the radiologist. The challenge with this opinion is the fact that AI systems are very much a “black box” for radiologists and radiographers who do not know how the systems come to develop these results let alone how their ability to provide a judgement. AI systems are developed to mimic the human brain; however, whilst human decisions are made logically based on obvious evidence, AI decisions are based on complex calculations that are often not revealed to humans and thus create an inability to explain their rationale. There are three types of AI programming, supervised, semi-supervised and unsupervised learning models. Semi- and un-supervised learning models are the most difficult ones as there is little knowledge about how they learn and thus analyse data and reach decisions. AI developers, although limited, are therefore the closest to explain how decisions are made as they are the ones who designed the algorithms and strategic direction to how the AI system should think. This also brings back the same problem of who will be the responsible when something goes wrong? This question perhaps will not be answered before further and more detailed investigations are conducted involving a wide range of stakeholders and supported with substantial evidence.

Despite the limited evidence, some researchers suggest that the application of AI can reduce the workload to approximately 53% which means moving from interpreting an image every 3–4 s (approximately 6,300 images per day) to 6–7 s (approximately 3,400 images per day). This is a good drop in workload which should reduce pressure, but perhaps the actual decision on whether this is good and reasonable timeframe or otherwise is to be determined by radiologists themselves. If the workload is still above the limits of radiologists, the use of AI would just be a delay of the problem rather than a resolution to it. Further investigation is needed to understand the capacity of radiologists over a period to develop a better understanding of their workload and how this can be standardised and most importantly how AI can help reduce their workload and burnout levels to enhance their retention and attraction.

The literature revealed that despite discussions that imply AI will replace radiologists, scientific evidence indicates that this is currently very difficult. Radiologists, radiographers and AI systems complement each other. AI systems can do repetitive operations, store and analyse substantial amounts of data to generate more comprehensive evidence to support radiologists in their decision making processes. Critically though, the data that is used by the AI algorithms needs to be robust; any errors introduced into the data could potentially train an AI system into persistently incorrect results. There is a possibility that jobs of radiologists and radiographers are ‘displaced’ to create more room for AI systems to do the repetitive tasks whilst new roles and jobs such as ‘radiomics’ and ‘radiogenomics’ develop. It is critical that any new such roles integrate radiologists so that medical care remains in the hands of the medically qualified. Software developers and other stakeholders must work with radiologists and be guided by them in the design of the ‘new standards’ to ensure that they define whether and where machine roles may be needed. Further research needs to be conducted to help collect radiologists, radiographers and relevant AI system developers views to identify areas for mutual collaboration. For example, the readiness of current radiology workforce to adopt AI as part of their daily routine taking into consideration their age and potentially their ethnic group. It is vital to emphasise the crucial role radiologists play in the provision of quality care in both normal day-to-day and during times of disaster and crisis. Their role in diagnosing and prognosing diseases provides guidance during preparedness, response and recovery [50] and helps patients receive the treatment they need without delay. The shortage of radiologists is a major sign of vulnerability of radiology services and thus needs to be addressed effectively.

### Ethical and legal frameworks

Despite the potential associated with the application of AI in radiology and support to mitigate the shortage of radiologists, there are significant challenges which are slowing down the implementation of this technology. Many hospital practices, management and staff are hesitant to accept and adopt the new technology because of ethical concerns such as respect for autonomy and confidentiality as well as legal concerns over liability should harm be caused, and fears for the future of the radiology profession and a distrust of AI by radiologists and the wider public. The findings established key ethical and legal concerns with the application of AI in radiology. These are related to risk/benefit, safety, failure and judicial transparency, privacy and staffing.

AI system misinterpretation of data is a concern that has been discussed in many studies despite the evidence presented to demonstrate the high sensitivity of new algorithms. Although AI in many cases is more accurate than humans in reading images and therefore should lead to greater patient safety overall, false negatives or positives can still lead to harm of patients, especially if there is an overreliance on the technology for decision making. It is also pertinent to note that AI makes its decisions based on data available to it. This data will not be representative of the entire population and hence any decisions are likely to be more accurate for some patients and less so for others. Ultimately AI is a series of algorithms, devoid of intuition. It would have great difficulty in interpreting unusual signs. If it makes a mistake, the liability might fall on the radiologist. Protecting radiologists from liability over mistakes made by AI is needed to increase confidence in embracing the technology. Concerns are also related to the potential bias associated with the reliance and dependency of radiologists on AI systems specifically at the stage where there is a little evidence of the risks associated with AI systems in the mid and long term. It is crucial that trainees are not taught to rely on AI but to interpret the images they see with any access to AI as they need to learn to develop their interpretive skills. Organisations need to ensure that these biases are minimised through ensuring that radiologists have the final decision at least in a transition period, by the end of which more evidence will be developed to support the way forward.

It is important to reach a consensus about who is held accountable for AI making critical mistakes that lead to the harm of a patient. The European Society of Radiology [51] demonstrated that there is a range of views as to who should be responsible for an error made by AI which confirms the findings of this review. Traditionally, the radiologist would be responsible for a clinical mistake. However, when AI has made the mistake, the issue of accountability is not clear.

Most of the studies focused their ethical and legal considerations on the use of data and information more than the protection of staff. Achour et al. [13] demonstrated that staff ability to attend the workplace depends on both professional and personal circumstances. These factors are equally important for staff members as they need to have assurance that they, and their families, are protected and safe. Having regulations, ethical policies that focus just on data and patients can indicate to staff that they are being overlooked and potentially under-minded.

### Comprehensiveness of literature and further development

Demographics of this study indicate that most of the publications (55%) are reviews. This denotes that there is a need for more primary work to help generate new knowledge and identify aspects related to the impact of AI on radiologists and radiographers. The findings show little diversity but too much consistency to the extent of ‘repetition’ which gives the impression of data saturation but could be due to the influence of research on each other. Comprehensive primary data research is needed to explore and provide a consensus of the application of AI in reducing the shortage of radiologists and radiographers and establish on whether AI represents a support or threat to their jobs or roles. Findings suggest that the generation of younger doctors are reluctant to develop careers in radiology, which suggests that current, older, generations who have less interaction with AI technology might have objections to using it. Understanding their views, feelings and readiness for such a challenge is important to understand how this technology can influence staff retention. In addition, understanding the views of young radiologists and radiographers will help identify their concerns and motivations. The wider and more diverse (age, cultural and economic backgrounds etc.) such a sample is, the better, richer and more representative the findings can be.

## Conclusions

This study explored the literature to collect evidence around the application of AI in radiology departments and the role they play in the shortage of radiologists. AI systems have developed high sensitivity to enable them to support decision making with high confidence. The literature suggests that AI can help in taking decisions for cases where the probability of illness is very high or low. This can lead to reducing the workload of radiologists to approximately 53%. However, there is a difference of opinions on the nature of this help and the trustworthiness of these systems due to ethical and legal issues mainly around the responsibility surrounding mistakes. AI systems Developers, radiologists and other stakeholders need to collaborate to develop systems that are able to meet expectations including staff concerns and those related to ethics and regulations.

In short, current evidence suggests that AI cannot replace radiologists, instead radiology services will need the input of radiologists, AI systems and radiographers to provide safe healthcare for all patients, therefore they are complementary. Radiologist jobs will most probably change to reduce some of the repetitive tasks to be conducted by AI. More research is needed to help identify this new change in addition to developing comprehensive evidence on the views of radiologists about their concerns and motivations to using AI and develop frameworks to support the multi-disciplinary team of stakeholders to work collaboratively in resolving the shortage of radiologists. No matter what this collaboration and relationship looks like, it is important to emphasise the crucial role radiologists and radiographers play in the provision of quality care in both normal day-to-day and during times of disaster emergency. Their role in diagnosing and prognosing diseases provides guidance during preparedness, response and recovery. The shortages of radiologists are a major sign of vulnerability of radiology services and thus needs to be addressed effectively.

## Data Availability

Not applicable.
